# Hippocampal‐sparing whole‐brain radiotherapy using the Elekta equipment

**DOI:** 10.1120/jacmp.v14i3.4205

**Published:** 2013-05-06

**Authors:** Alexander Nevelsky, Nantakan Ieumwananonthachai, Orit Kaidar‐Person, Raquel Bar‐Deroma, Haitam Nasrallah, Rahamim Ben‐Yosef, Abraham Kuten

**Affiliations:** ^1^ Division of Oncology Rambam Health Care Campus Haifa Israel; ^2^ Chulabhorn Cancer Centre Bangkok Thailand

**Keywords:** brain metastases, conformal avoidance, hippocampus, linear accel erator, radiotherapy, whole brain

## Abstract

The purpose of this study was to evaluate the feasibility of hippocampal‐sparing whole‐brain radiotherapy (HS WBRT) using the Elekta Infinity linear accelerator and Monaco treatment planning system (TPS). Ten treatment plans were created for HS‐WBRT to a dose of 30 Gy (10 fractions). RTOG 0933 recommendations were applied for treatment planning. Intensity‐modulated radiotherapy (IMRT) plans for the Elekta Infinity linear accelerator were created using Monaco 3.1 TPS‐based on a nine‐field arrangement and step‐and‐shoot delivery method. Plan evaluation was performed using D2% and D98% for the whole‐brain PTV (defined as whole brain excluding hippocampus avoidance region), D100% and maximum dose to the hippocampus, and maximum dose to optic nerves and chiasm. Homogeneity index (HI) defined as $(D2\%-D98\%)/D_{median}$ was used to quantify dose homogeneity in the PTV. The whole‐brain PTV D2% mean value was 37.28 Gy (range 36.95–37.49 Gy), and D98% mean value was 25.37 Gy (range 25.40–25.89 Gy). The hippocampus D100% mean value was 8.37 Gy (range 7.48–8.97 Gy) and the hippocampus maximum dose mean value was 14.35 Gy (range 13.48–15.40 Gy). The maximum dose to optic nerves and optic chiasm for all patients did not exceed 37.50 Gy. HI mean value was 0.36 (range 0.34–0.37). Mean number of segments was 105 (range 88–122) and mean number of monitor units was 1724 (range 1622–1914). Gamma evaluation showed that all plans passed 3%, 3 mm criteria with more than 99% of the measured points. These results indicate that Elekta equipment (Elekta Infinity linac and Monaco TPS) can be used for HS WBRT planning according to compliance criteria defined by the RTOG 0933 protocol.

PACS numbers: 87.55D, 87.55 –v, 87.55 de

## I. INTRODUCTION

In the past, whole‐brain radiotherapy (WBRT) planning was simple. Today, new clinical and dosimetric considerations are taken into consideration when approaching such planning. It has been found that as many as 11% of patients who were treated by WBRT and survived more than 12 months developed dementia, especially with the use of a larger dose‐per‐fraction regimen.^(1)^ However, regression of the lesions after WBRT was found to correlate with survival and improved neurocognitive function. Therefore, achievement of macroscopic lesion control is the mainstay of treatment. This is also supported by reports that the median time to neurocognitive function deterioration was longer in patients whose disease regressed after WBRT than in poor responders.^(2)^ Thus, treatment‐dose compromise is unjust for preserving these neurocognitive functions. Furthermore, memory functions were found to be most susceptible to early decline, even in patients with nonprogressing brain metastases.^(2)^ These concerns became more significant as WBRT was instituted for prophylactic brain irradiation (PCI) for various neoplasms to decrease intracranial failure in patients with potential long‐term survival.^(3)^

The current postulate is that, although several functional areas of the brain are responsible for neurocognitive functions, the stem cells residing within the subgranular zone of the hippocampus are necessary for neurogenesis, especially for the memory domains. Radiation induces inflammation in the microenvironment which causes depletion of these cells, resulting in deterioration in functions of learning, memory (short and long‐term), and spatial information processing.^(4)^ Hippocampus sparing during WBRT would potentially reduce neurocognitive deficits caused by radiation. A study that evaluated the distribution of brain metastases with relation to the hippocampus found that, of 100 patients with a total of 272 brain metastases, eight patients had lesions 5 mm from the hippocampus. Eighty‐six percent (n=235) of metastases were at a distance greater than 15 mm from the hippocampus.^(5)^ Similar results were evident in the RTOG 0933 phase II clinical trial^(6)^ which indicated that, of 1133 metastases in 371 patients, only 8.6% metastases were within 5 mm of the hippocampus. None of the metastases were present within the hippocampus. These results suggest that hippocampal‐sparing WBRT (HS WBRT) does not compromise treatment.^(7)^

The anatomic shape and central location of the hippocampus poses a challenge for contouring and treatment planning. Several publications have reported on techniques for HS WBRT using tomotherapy^8^, ^9^, ^10^ and Varian equipment.^8^, ^11^ Based on these publications, the RTOG 0933 protocol suggested criteria for HS WBRT plan evaluation and acceptance. Moreover, RTOG 0933 recommended approaches to using helical tomotherapy and linac‐based IMRT planning, including beam arrangements and a set of inverse‐planning algorithm constraints which were found to meet the dosimetric criteria set in the protocol.

The purpose of this work was to evaluate the feasibility of HS WBRT using the Elekta Infinity linear accelerator and the Monaco treatment planning system (TPS) according to RTOG compliance criteria, and to offer recommendations for HS WBRT using this Elekta equipment.

## II. MATERIALS AND METHODS

Ten patients previously treated by WBRT underwent repeated planning in an attempt to spare the hippocampus region. Magnetic resonance imaging (MRI) and computerized tomography (CT) sets were fused for each patient prior to delineation of the hippocampus, optic nerves, and chiasm. The hippocampus avoidance region was generated by expanding the hippocampus volumetrically by 5 mm. RTOG 0933 recommendations^(6)^ (Table 1) were applied for treatment planning. The whole‐brain dose prescription was 30 Gy in 10 fractions, and the whole‐brain PTV was defined as the whole brain excluding the hippocampus avoidance region.

**Table 1:** RTOG 0933 compliance criteria.

*Organ*	*Dose Constraints*
Whole‐brain PTV	D2%<37.5 Gy (D2%<40Gy is allowed)
	D98%>25 Gy
	V30>30 Gy
Hippocampus	D100%<9 Gy
	$D_{\max}<16\;Gy$ ($D_{\max}<17\;Gy$ is allowed)
Maximum dose to optic chiasm and optic nerves	37.5 Gy

IMRT plans for the Elekta Infinity linear accelerator (Elekta AB, Stockholm, Sweden) were created using Monaco 3.1 (Elekta AB) TPS based on a nine‐field arrangement and step‐and‐shoot delivery method. The Elekta Infinity system is equipped with MLCi2 multileaf collimator (MLC), which has 40 leaf pairs, each with a width of 1 cm and a minimum gap of 0.5 cm at the isocenter, and is capable of delivering volumetric‐modulated arc therapy (VMAT) plans. A detailed description of the Monaco optimization cost functions and algorithm was given by Semenenko et al.^(12)^ Briefly, in addition to the standard physical cost functions found with major treatment planning systems, Monaco offers one objective biological cost function for the target and two constraint biological cost functions based on a general parallel or serial model that can be used to shape the dose distribution. Unlike dose‐based constraints which act only upon a single point on the dose‐volume histogram (DVH), the biological cost functions act across the entire DVH. The optimization process is divided into two stages. During the first stage, the ideal dose fluence map is optimized based on the beam parameters and the optimization constraints. At that stage, dose distribution is computed using a pencil beam algorithm. During the second stage, the MLC sequencer performs the segmentation, which includes optimization of segment shapes and weights, so that deliverable fields are obtained. For each field, the dose distribution is recalculated using a Monte Carlo algorithm based on the corresponding MLC segments, and then reoptimized as necessary. Details on validation of the algorithms are reported in several publications.^13^, ^14^, ^15^

The Monaco TPS has two optimization modes: constrained optimization and Pareto optimization mode. The constrained optimization sets equally weighted constraints on healthy tissues which must be met while the dose‐to‐target is administered. Usually, more than one dose distribution can achieve the same biological effect, and the optimizer has more flexibility to shape the dose than when strict physical and often contradictory constraints are used. The constrained optimization works very effectively in the vast majority of clinical situations and is used as the default optimization mode for Monaco. The Pareto mode effectively reverses how the Monaco normally works. During optimization, Monaco relaxes constraints on healthy tissues enough to satisfy the underdose requirements on the target volumes. HS WBRT represents a very rare clinical case when the Pareto mode has to be used to assure the target coverage according to compliance criteria set by RTOG protocol. The beam arrangements suggested by the RTOG protocol include seven or eight couch angles. In order to reduce treatment time by reducing the number of couch angles on the one hand, and to assure compliance with RTOG criteria on the other hand, we devised an alternative nine‐field arrangement which included seven coplanar and two noncoplanar beams. Details of the field arrangement are presented in Table 2.

For beams from one to seven, the collimator was angled to 45° so that the leaves are differentially oriented with respect to the patient throughout the gantry rotation, thereby improving spatial resolution of MLC. Selection of plan segmentation parameters was found to be a crucial factor for efficient segmentation and final plan quality. In Table 3 we report a set of segmentation parameters which produced plans of acceptable quality with a reasonable number of segments. This set was successfully applied for all ten patients in the study.

**Table 2:** Beam arrangement used in our study.

*Beam*	*Gantry Angle*	*Collimator Angle*	*Couch Angle*
1	10	45	0
2	60	45	0
3	130	45	0
4	170	45	0
5	220	45	0
6	270	45	0
7	320	45	0
8	290	0	90
9	330	0	90

**Table 3:** Set of segmentation parameters which produced plans of acceptable quality with reasonable number of segments.

Calculation grid	0.2 cm
Monte Carlo variance per plan	1%
Minimum segment area	4 cm
Minimum segment change area	4 cm
Minimum segment width	0.5 cm
Minimum MU/segment	6 MU

Plan evaluation was performed using D2% and D98% for the whole‐brain PTV, D100% and maximum dose to the hippocampus, and maximum dose to optic nerves and chiasm. Homogeneity index (HI) defined as $(D2\%-D98\%)/D_{meadian}$, according to recommendations of the International Commission on Radiation Units and Measurements,^(16)^ was used to quantify dose homogeneity in the PTV.

Quality assurance (QA) of the plan delivery was performed by measuring the dose distribution using a ScandiDos rotational diode‐array phantom, Delta^4^ (ScandiDos, Uppsala, Sweden). The Delta^4^ dosimeter consists of 1069 p‐type silicone diodes 0.78 mm^2^ in size. The detectors are embedded into boards that form two orthogonal planes within a polymethylmethacrylate (PMMA) cylindrical phantom 22 cm in diameter. The “main” detector plane has an active area of $20\times 20\;{cm}^{2}$ with diodes placed 5 mm apart within a central $6\times 6\;{cm}^{2}$ region and 10 mm apart elsewhere. The second orthogonal plane is split into two “wings” that have an area of $20\times 10\;{cm}^{2}$ each, with the same overall detector spacing. Further details on the Delta^4^ design and performance can be found in previous publications.^17^, ^18^, ^19^ As one of the noncoplanar beams in our beam arrangement (couch angle 90° and gantry angle 290°) would pass through the top edge of the Delta^4^ cylindrical phantom, we reset all beams to one gantry angle of 0° for the QA calculations and measurements. The Monte Carlo calculation uncertainty and the calculation grid size for QA plans were set to 1% and 3 mm. The experimental Delta^4^ dose distributions were compared to the Monaco TPS calculations using the γ criteria^(20)^ with two sets of dose difference/distance‐to‐agreement parameters: clinical 3%/3 mm and more stringent 2%/2 mm set. Beam‐on time was recorded during the QA measurements.

## III. RESULTS

**Figure 1:**
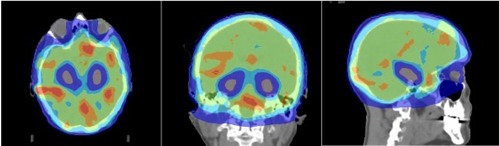
Dose distribution (axial, coronal, and sagittal views) at the level of the hippocampi for hippocampal avoidance during whole‐brain radiotherapy for one representative patient. Red contour represents the hippocampus. Blue isodose represents 12 Gy; light blue, 25 Gy; green, 30 Gy; orange, 35 Gy; red, 37.5 Gy, in ten fractions.

**Figure 2:**
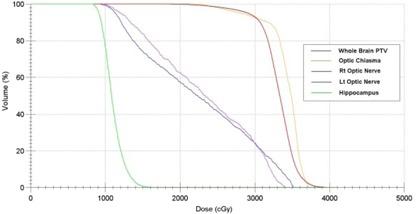
A cumulative normalized dose‐volume histogram for whole‐brain radiotherapy with hippocampal sparing for one representative patient.

Dose distribution (axial, coronal, and sagittal views) for one representative patient is shown in Fig. 1 and a dose‐volume histogram (DVH) for one representative patient is shown in Fig. 2.

The whole‐brain PTV D2% mean value was 37.28 Gy (range 36.95–37.49 Gy), and D98% mean value was 25.37 Gy (range 25.40–25.89 Gy). The PTV V30 mean value was 92% (range 90.5%–93.2%). The hippocampus D100% mean value was 8.37 Gy (range 7.48–8.97 Gy), and the maximum dose mean value was 14.35 Gy (range 13.48–15.40 Gy). The maximum dose to optic nerves and optic chiasm for all patients did not exceed 37.50 Gy. HI mean value was 0.36 (range 0.34–0.37). Detailed dosimetric results for ten plans are presented in Table 4.

Mean number of segments was 105 (range 88–122), and mean number of monitor units (MU) was 1724 (range 1622–1914). Using the clinical gamma criteria, 3% and 3 mm, the mean passing rate (gamma <1) was 99.4%. Using gamma criteria 2% and 2 mm, the mean passing rate was 93.5%. The average beam‐on time was 13 min 27 sec. Detailed information on number of segments, number of MUs, and gamma values is presented in Table 5.

**Table 4:** Detailed dosimetric results for ten patients.

*Pt. No.*	*PTV D2% (Gy)*	*PTV D98% (Gy)*	*V30 (%)*	*HC D100% (Gy)*	*HC HC Max (Gy)*	*Opt Nerve Max (Gy)*	*Opt Chiasm Max (Gy)*	*HI*
1	36.98	25.58	93.2	7.72	15.21	29.62	36.44	0.34
2	37.41	25.74	92.8	8.62	15.75	35.30	36.60	0.36
3	36.94	25.87	93.0	8.03	15.38	29.95	36.50	0.34
4	37.57	25.72	91.8	8.82	15.94	30.90	34.49	0.36
5	37.22	25.57	92.0	8.48	15.16	34.84	36.66	0.37
6	36.98	25.40	90.7	8.92	15.49	34.62	35.49	0.37
7	37.22	25.89	93.2	8.82	15.74	34.08	36.50	0.37
8	37.50	25.65	90.5	8.87	14.28	35.74	33.94	0.37
9	36.96	25.61	91.6	7.68	16.06	31.67	37.15	0.36
10	36.93	25.69	92.0	7.83	14.87	28.27	36.00	0.37

**Table 5:** Detailed information on number of segments, number of MUs, and gamma values for ten patients.

*Pt. No.*	*Segments*	*MU*	*Beam‐on Time (min)*	*Gamma* 2/2 (%)	*Gamma* 3/3 (%)
1	104	1644	13:05	93.9	99.5
2	88	1640	11:48	93.4	99.2
3	122	1675	14:32	93.2	99.2
4	95	1640	12:18	93.3	99.4
5	119	1854	14:52	93.5	99.5
6	109	1914	14:11	93.5	99.4
7	106	1692	13:24	93.3	99.3
8	90	1622	11:56	93.5	99.3
9	109	1807	13:46	93.8	99.6
10	120	1750	14:42	93.4	99.2

## IV. DISCUSSION

The most important factor in successful HS WBRT planning is the TPS. Our results show that the Monaco TPS allows creation of plans that fully comply with RTOG 0933 criteria. Apart from the RTOG criteria, there are several other parameters which help to evaluate the plans in terms of dosimetric quality, deliverability, and effectiveness. These parameters include HI, beam‐on time, number of MUs, and gamma value. Some of these parameters were reported in other publications and can be compared to our results.

Gutiérrez et al.^(9)^ studied HS WBRT with simultaneous integrated boost for brain metastases using helical tomotherapy. The brain dose prescription was 32.25 Gy in 15 fractions, while the boost doses to individual brain metastases were 63 Gy or 70.8 Gy, depending on the lesion diameter. Gutiérrez and colleagues reported HI ranging between 0.32 and 0.48, depending on the tomotherapy pitch and the field width (FW); this is comparable to mean HI of 0.36 in our results. They also reported a mean treatment time of 10 min for 2.5 cm FW plans and 21.8 min for 1.0 cm FW plans. RTOG 0933 recommends 1.0 cm FW for the HS WBRT using tomotherapy.

Marsh et al.^(10)^ also used helical tomotherapy in their study aimed on the hippocampus sparing during WBRT or prophylactic cranial irradiation (PCI). Dose prescription was 35 Gy in 14 fractions (2.5 Gy per fraction) for WBRT plans and 30 Gy in 15 fractions (2 Gy per fraction) for PCI plans. Mean PCI and WBRT treatment times were essentially identical (mean 15.23 min, range 14.27–17.5) in their study. As the mean beam‐on time measured for our plans was 13.5 min (about 1 min has to be added for a real treatment time as we did not rotate the couch to 270° during QA measurements), we can see that the delivery time in our study was comparable to that reported in publications of Gutiérrez and Marsh where helical tomotherapy was used.

Hsu et al.^(11)^ used VMAT technique for HS WBRT with simultaneous integrated boost for brain metastases; dose prescription was the same as used in Gutiérrez's paper. Hsu and colleagues were able to deliver plans in 3–4 min on a Varian CL21 EX linear accelerator with a Millennium 120‐leaf MLC (Varian Medical Systems, Palo Alto, CA). This is very impressive result, compared to the treatment times discussed above. They reported mean HI of 0.39, which is comparable to our results. It should be noted, however, that in their study the in‐house VMAT optimization planning system environment was used. This environment is based on the VMAT optimization algorithm published by Otto.^(21)^ Dose calculations were performed using a pencil beam algorithm^(11)^ (see p. 1481). It is known^22^, ^23^, ^24^ that, with this algorithm, it is easier to achieve optimization objectives than with more advanced algorithms such as Monte Carlo, due to limitations of the penumbra and 3D scatter modeling and, as a result, more optimistic DVH for target coverage, homogeneity, and OAR sparing.

Gondi et al.^(8)^ performed a planning study for HS WBRT utilizing tomotherapy and linac‐based IMRT. Dose prescription was 30 Gy in ten fractions. They reported mean HI of 0.16 using helical tomotherapy and HI of 0.30 using Varian linear accelerator. For the linac‐based HS WBRT, the optimized beam arrangement was generated using the Varian Plan Geometry Optimizer; this arrangement included nine noncoplanar beams with seven different couch angles. For the treatment planning, Pinnacle^3^ version 8.0m TPS (Philips Healthcare, Andover, MA) was used. In the Gondi study, no MUs or treatment time were reported; however, one can assume that the beam arrangement used for their plans with different couch angles would not allow rapid treatment delivery. No quality assurance (QA) procedures for the produced plans were performed, so the question of deliverability and calculation accuracy remained open in this work.

Hsu et al.^(11)^ performed QA procedures for all treatment plans; they showed that the measured dose at isocenter agreed with the planned dose within 1.8±0.8. The dose distributions in axial, coronal, and sagittal planes were measured using radiographic films. The results of these measurements showed good correlation between calculated and measured doses; however, no gamma analysis results were reported. Gutiérrez et al.^(9)^ and Marsh et al.^(10)^ performed patient‐specific QA for one plan only. The Gutiérrez study reported good correlation between calculated and measured dose distributions without specifying the gamma criteria passing rate.

The Marsh study found about 90% passing rate for gamma criteria with 3%/3 mm. It was the only study where the passing rate for gamma analysis was reported.

Our results of gamma analysis show more than 99% of the points pass the 3%, 3 mm gamma criteria, and more than 93% of the points pass for the 2%, 2 mm criteria for all ten plans. Based on these results, we conclude that dose distributions calculated with Monaco 3.1 for the HS WBRT are in very good agreement with the measured doses.

Since a simple beam arrangement based on nine equally spaced coplanar fields did not allow for obtaining good results, we devised a field setup based on seven coplanar and two noncoplanar beams, with gantry angles selected to spare the hippocampus more effectively and keep the treatment time reasonable. This beam arrangement, as well as segmentation and dose calculation parameters, was saved as a template. Although we cannot claim that this template represents the best treatment setup, it worked well for all ten treatment plans.

All IMRT plans were based on the step‐and‐shot delivery method. It is worth mentioning that, due to sequencer limitation in the current version of Monaco, we were unable to produce VMAT plans fully complying with RTOG criteria. As the VMAT technique can potentially reduce delivery time, further studies are needed to evaluate the use of VMAT for HS WBRT treatment with the newer (above 3.1) versions of Monaco TPS, and to compare its quality with step‐and‐shoot IMRT plans.

There are also physical factors that could influence the quality of plans for HS WBRT. Kirby et al.^(25)^ concluded that intracranial scattering alone is responsible for a large dose contribution to the stem cell compartment. Therefore, it is important to minimize other contributing factors, particularly collimator leakage, to maximally reduce the dose to these critical structures. In addition, penumbra size, which defines the steepness of the dose falloff around the hippocampus‐avoidance region, and the MLC leaf width, which determines spatial resolution of dose distribution, may also be important factors influencing plan quality. These characteristics depend on the design of the specific MLC.

Huq et al.^(26)^ investigated dosimetric characteristics of MLCs with different designs and found that the Elekta MLC has a larger penumbra (about 6 mm) than the Varian MLC (about 4 mm). This is due to the fact that the Varian MLC is placed as a tertiary system below the standard adjustable jaws and is much closer to the patient than the Elekta system, which replaces the upper jaw of the standard collimator. Also, interleaf leakage was slightly higher for the Elekta MLC (about 2.5%) compared to the Varian MLC (about 1.8%). Both the Elekta and Varian MLCs have additional backup jaws that travel in the same direction as the MLC. However, in the Elekta MLC design, the backup jaws are programmed to align always with the most retracted leaf. Using the backup jaw together with the leaves allows bringing the leakage radiation down to 0.5%, and, by letting the backup jaws follow each IMRT segment, it is possible to achieve a lower overall radiation leakage for the Elekta MLC than for the Varian MLC. In summary, the linac design can affect the quality of plans for HS WBRT. We show that it is possible to create HS WBRT plans compliant with the requirements of RTOG 0933 protocol using Elekta Infinity machine with 1 cm MLC.

## V. CONCLUSIONS

The current study shows the feasibility of planning HS WBRT using Elekta equipment (Monaco TPS and Elekta Infinity linac) according to the very demanding compliance criteria defined by the RTOG 0933 protocol, and provides planning recommendations which can be used by Elekta equipment users, in addition to those given in the RTOG protocol.

## ACKNOWLEDGMENTS

The authors would like to thank Irina Fotina, PhD, of Elekta, for the valuable remarks and productive technical discussions.
